# Adjuvant capecitabine in triple negative breast cancer patients with residual disease after neoadjuvant treatment: real-world evidence from *CaRe*, a multicentric, observational study

**DOI:** 10.3389/fonc.2023.1152123

**Published:** 2023-05-16

**Authors:** Francesca Sofia Di Lisa, Eriseld Krasniqi, Laura Pizzuti, Maddalena Barba, Katia Cannita, Ugo De Giorgi, Fulvio Borella, Jennifer Foglietta, Anna Cariello, Antonella Ferro, Elisa Picardo, Marco Mitidieri, Valentina Sini, Simonetta Stani, Giuseppe Tonini, Daniele Santini, Nicla La Verde, Anna Rita Gambaro, Antonino Grassadonia, Nicola Tinari, Ornella Garrone, Giuseppina Sarobba, Lorenzo Livi, Icro Meattini, Giuliana D’Auria, Matteo Vergati, Teresa Gamucci, Mirco Pistelli, Rossana Berardi, Emanuela Risi, Francesco Giotta, Vito Lorusso, Lucia Rinaldi, Salvatore Artale, Marina Elena Cazzaniga, Fable Zustovich, Federico Cappuzzo, Lorenza Landi, Rosalba Torrisi, Simone Scagnoli, Andrea Botticelli, Andrea Michelotti, Beatrice Fratini, Rosa Saltarelli, Ida Paris, Margherita Muratore, Alessandra Cassano, Lorenzo Gianni, Valeria Gaspari, Enzo Maria Veltri, Federica Zoratto, Elena Fiorio, Maria Agnese Fabbri, Marco Mazzotta, Enzo Maria Ruggeri, Rebecca Pedersini, Maria Rosaria Valerio, Lorena Filomeno, Mauro Minelli, Paola Scavina, Mimma Raffaele, Antonio Astone, Roy De Vita, Marcello Pozzi, Ferdinando Riccardi, Filippo Greco, Luca Moscetti, Monica Giordano, Marcello Maugeri-Saccà, Alessandro Zennaro, Claudio Botti, Fabio Pelle, Sonia Cappelli, Flavia Cavicchi, Enrico Vizza, Giuseppe Sanguineti, Federica Tomao, Enrico Cortesi, Paolo Marchetti, Silverio Tomao, Iolanda Speranza, Isabella Sperduti, Gennaro Ciliberto, Patrizia Vici

**Affiliations:** ^1^ Phase IV Clinical Studies Unit, IRCCS Regina Elena National Cancer Institute, Rome, Italy; ^2^ Division of Medical Oncology 2, IRCCS Regina Elena National Cancer Institute, Rome, Italy; ^3^ Oncology Division, Mazzini Hospital, ASL Teramo, Teramo, Italy; ^4^ Department of Medical Oncology, IRCCS Istituto Romagnolo per lo Studio dei Tumori (IRST) “Dino Amadori”, Meldola, Italy; ^5^ Gynecology and Obstetrics 1, Department of Surgical Sciences, City of Health and Science, Sant' Anna Hospital, University of Turin, Turin, Italy; ^6^ Medical Oncology, P.O. Narni-Amelia, Terni, Italy; ^7^ Oncology Department, AUSL Romagna, Ravenna, Italy; ^8^ Breast Center, Santa Chiara Hospital, Trento, Italy; ^9^ Gynecology and Obstetrics 4, Department of Surgical Sciences, City of Health and Science, Sant' Anna Hospital, University of Turin, Turin, Italy; ^10^ Medical Oncology, Santo Spirito Hospital, Rome, Italy; ^11^ Department of Medical Oncology, Fondazione Policlinico Universitario Campus Biomedico, Rome, Italy; ^12^ Medical Oncology A, Policlinico Umberto I, Department of Radiological, Oncological and Anatomo-Pathological Sciences, “Sapienza” University of Rome, Rome, Italy; ^13^ Department of Oncology, Ospedale Luigi Sacco, ASST Fatebenefratelli Sacco, Milan, Italy; ^14^ Department of Innovative Technologies in Medicine and Dentistry and Centre for Advanced Studies and Technology (CAST), G. D’Annunzio University, Chieti, Italy; ^15^ Department of Medical, Oral and Biotechnological Sciences and Center for Advanced Studies and Technology (CAST), G. D’Annunzio University, Chieti, Italy; ^16^ Medical Oncology, Fondazione IRCCS Ca’Granda Ospedale Maggiore Policlinico Milano, Milan, Italy; ^17^ Department of Medical Oncology, ASL Nuoro, Nuoro, Italy; ^18^ Department of Biomedical, Experimental and Clinical Sciences “M. Serio”, University of Florence, Florence, Italy; ^19^ Radiotherapy Unit, Oncology Department, Azienda Ospedaliera Universitaria Careggi, Florence, Italy; ^20^ UOC of Medical Oncology, Sandro Pertini Hospital, Rome, Italy; ^21^ Oncology Clinic, Università Politecnica delle Marche, Ospedali Riuniti Hospital, Ancona, Italy; ^22^ Sandro Pitigliani Medical Oncology Department, Hospital of Prato, Prato, Italy; ^23^ Department of Medical Oncology, IRCCS Giovanni Paolo II Institute, Bari, Italy; ^24^ “Don Tonino Bello” Oncology Unit, IRCCS Istituto Tumori “Giovanni Paolo II”, Bari, Italy; ^25^ Oncology Department, Ospedale di Gallarate, ASST Valle Olona, Gallarate, Italy; ^26^ Phase 1 Research Centre and Oncology Unit, Department of Medicine and Surgery, University of Milano-Bicocca, ASST Monza, Monza, Italy; ^27^ Oncology Unit, ASST Monza, Monza, Italy; ^28^ Oncology Division, AULSS 1 Dolomiti, San Martino Medical Hospital, Belluno, Italy; ^29^ Phase I Clinical Studies Unit, IRCCS Regina Elena National Cancer Institute, Rome, Italy; ^30^ Department of Medical Oncology and Hematology Unit, IRCCS Humanitas Research Hospital, Milan, Italy; ^31^ Department of Medical and Surgical Sciences and Translational Medicine, “Sapienza” University of Rome, Rome, Italy; ^32^ UO Medical Oncology I, S. Chiara Hospital, Pisa University Hospital, Pisa, Italy; ^33^ Oncology Division, San Giovanni Evangelista Hospital, ASL RM5, Rome, Italy; ^34^ Division of Gynecologic Oncology, Department of Woman and Child Health and Public Health, Fondazione Policlinico Universitario Agostino Gemelli IRCCS, Rome, Italy; ^35^ Medical Oncology, Comprehensive Cancer Center, Fondazione Policlinico Universitario Agostino Gemelli IRCCS, Università Cattolica del Sacro Cuore, Rome, Italy; ^36^ Oncology Unit Rimini, Azienda USL Romagna, Rimini, Italy; ^37^ Medical Oncology Unit, Santa Maria Goretti Hospital, Latina, Italy; ^38^ Pathology Unit, Azienda Ospedaliera Universitaria Integrata, Verona, Italy; ^39^ Medical Oncology Unit, Belcolle Hospital, Viterbo, Italy; ^40^ Breast Unit-Oncologia, ASST-Spedali Civili, Brescia, Italy; ^41^ Medical Oncology, Azienda Ospedaliera Universitaria Policlinico Paolo Giaccone, Palermo, Italy; ^42^ Division of Oncology, San Giovanni Hospital, Rome, Italy; ^43^ Presidio Oncologico Cassia – S. Andrea, ASL Roma 1, Rome, Italy; ^44^ Oncology Division, San Pietro Fatebenefratelli Hospital, Rome, Italy; ^45^ Department of Plastic and Reconstructive Surgery, IRCCS Regina Elena National Cancer Institute, Rome, Italy; ^46^ Oncology Unit, Antonio Cardarelli Hospital, Naples, Italy; ^47^ Medical Oncology Unit, AULSS 9 Regione Veneto, Scaligera - Ospedale Generale Mater Salutis, Legnago, Italy; ^48^ Division of Medical Oncology, Department of Oncology-Hematology, University Hospital of Modena, Modena, Italy; ^49^ Medical Oncology Division, ASST-Lariana, Como, Italy; ^50^ Clinical Trial Center, Biostatistics and Bioinformatics, IRCCS Regina Elena National Cancer Institute, Rome, Italy; ^51^ Department of Surgery, IRCCS Regina Elena National Cancer Institute, Rome, Italy; ^52^ Gynecologic Oncology Unit, IRCCS Regina Elena National Cancer Institute, Rome, Italy; ^53^ Department of Radiation Oncology, IRCCS Regina Elena National Cancer Institute, Rome, Italy; ^54^ Department of Maternal and Child Health and Urological Sciences, “Sapienza” University of Rome, Policlinico Umberto I, Rome, Italy; ^55^ Medical Oncology B, Policlinico Umberto I, Department of Radiological, Oncological and Pathological Sciences, “Sapienza” University of Rome, Rome, Italy; ^56^ Scientific Direction, IRCCS IDI, Istituto Dermopatico dell'Immacolata, Rome, Italy; ^57^ Department of Radiological, Oncological and Pathological Sciences, “Sapienza” University of Rome, Rome, Italy; ^58^ Scientific Direction, IRCCS Regina Elena National Cancer Institute, Rome, Italy

**Keywords:** triple negative breast cancer, neoadjuvant treatment, residual tumors, adjuvant capecitabine, treatment discontinuation

## Abstract

**Background:**

In triple negative breast cancer patients treated with neoadjuvant chemotherapy, residual disease at surgery is the most relevant unfavorable prognostic factor. Current guidelines consider the use of adjuvant capecitabine, based on the results of the randomized *CREATE-X* study, carried out in Asian patients and including a small subset of triple negative tumors. Thus far, evidence on Caucasian patients is limited, and no real-world data are available.

**Methods:**

We carried out a multicenter, observational study, involving 44 oncologic centres. Triple negative breast cancer patients with residual disease, treated with adjuvant capecitabine from January 2017 through June 2021, were recruited. We primarily focused on treatment tolerability, with toxicity being reported as potential cause of treatment discontinuation. Secondarily, we assessed effectiveness in the overall study population and in a subset having a minimum follow-up of 2 years.

**Results:**

Overall, 270 patients were retrospectively identified. The 50.4% of the patients had residual node positive disease, 7.8% and 81.9% had large or G3 residual tumor, respectively, and 80.4% a Ki-67 >20%. Toxicity-related treatment discontinuation was observed only in 10.4% of the patients. In the whole population, at a median follow-up of 15 months, 2-year disease-free survival was 62%, 2 and 3-year overall survival 84.0% and 76.2%, respectively. In 129 patients with a median follow-up of 25 months, 2-year disease-free survival was 43.4%, 2 and 3-year overall survival 78.0% and 70.8%, respectively. Six or more cycles of capecitabine were associated with more favourable outcomes compared with less than six cycles.

**Conclusion:**

The *CaRe* study shows an unexpectedly good tolerance of adjuvant capecitabine in a real-world setting, although effectiveness appears to be lower than that observed in the *CREATE-X* study. Methodological differences between the two studies impose significant limits to comparability concerning effectiveness, and strongly invite further research.

## 1 Introduction

The triple negative subtype accounts for about 15% of breast cancers, and is usually associated with poor outcomes (1). Due to the lack of hormonal and human epidermal growth factor receptor 2 (HER2), the only widely reknown therapeutic strategy is systemic chemotherapy, which still remains the mainstay of treatment (2). Despite the advances in neoadjuvant/adjuvant chemotherapy regimens, about 25-35% of the patients develop metastatic disease, more often within 5 years from diagnosis (3, 4).

Notwithstanding the unfavourable prognosis, triple negative breast cancer is considered highly sensitive to chemotherapy. Neoadjuvant treatment is commonly employed in this subset of patients, since it increases the rate of conservative surgery. Most importantly, neoadjuvant chemotherapy may determine the achievement of pathological complete response (pCR), which is related to significantly better long-term outcomes (5, 6). Unfortunately, approximately half of the patients with triple negative breast cancer treated with standard neoadjuvant anthracyclines and taxanes do not achieve pCR, showing residual disease in the breast and/or axilla at surgery. This subset of patients has an unfavorable prognosis, with high risk of recurrence (7).

Several efforts have been made to improve prognosis in these patients, and post-neoadjuvant strategies with no cross resistant or new agents have been recently evaluated, particularly in the HER2 positive and triple negative subtypes (8). Some studies tested additional therapies in the post-neoadjuvant setting of triple negative tumors, without well defined treatments recommended for patients with residual disease at definite surgery. Among the chemotherapic agents, those more commonly studied in the triple negative subtype have been capecitabine and platinum salts.

Capecitabine, a pro-drug converted into fluorouracil, is an orally available agent largely employed in breast cancer patients, mainly in the advanced setting. Considering post-neoadjuvant strategies, first data come from the *Capecitabine for Residual Cancer as Adjuvant Therapy (CREATE-X)* (9), a randomized phase III trial enrolling patients with stage I-III, HER2 negative breast cancer not achieving a pathological complete response to neoadjuvant treatment. Eight-hundred and eighty-seven patients were randomly assigned to follow-up or oral capecitabine (1,250 mg/m2 twice daily on days 1-14 every 3 weeks for 8 cycles). All patients in both arms received endocrine therapy and/or radiotherapy if indicated according to standard guidelines. At final analysis, overall, the 5 year (yr) disease free survival (DFS) was 67.6% in the control arm and 74.1% in the capecitabine arm (HR) 0.70, p=0.01). The 5yr overall survival (OS) was 83.6% versus 89.2% (HR 0.59, p=0.01). The most impressive results were observed in the triple negative subtype (N: 286 patients), wherein DFS-related HR was 0.50, and OS related HR was 0.52. The safety profile of capecitabine in the *CREATE-X* study was consistent with previous findings, but the trial was conducted exclusively in Japanese and Korean patients, with possibly pharmacogenomics and pharmacokinetic differences in drug metabolism as compared with Western populations (9–12).

The results of the *CREATE-X* study are certainly relevant, particularly for the triple negative subtype, the breast cancer patients’ subgroup with the most unfavorable outcomes, for which no effective adjunctive treatments have been established so far.

Based on the results of the above reported trial, recent guidelines have now considered the use of adjuvant capecitabine in triple negative patients with residual disease at surgery after neoadjuvant treatment. To date, no extensive data are available on the use of adjuvant capecitabine in real-world setting outside of clinical trials, and in Caucasian patients. On this basis, we carried out a multicenter, observational, retrospective study to evaluate the tolerability and, secondarily, the effectiveness outcomes of adjuvant capecitabine in patients with triple negative breast cancer treated with neoadjuvant chemotherapy and having invasive residual disease at surgery.

## 2 Materials and methods

### 2.1 Study approval

The “Adjuvant *Ca*pecitabine in triple negative breast cancer patients with *Re*sidual disease after neoadjuvant treatmentReal-world evidence from *CaRe*, a multicentric, observational study”, is a multicenter, observational, retrospective study recruiting triple negative early breast cancer patients with invasive residual disease at surgery after neoadjuvant chemotherapy including antracyclines and/or taxanes or platinum-derivatives.

The study was approved by the Institutional Review Board (IRB) of the coordinating centre, the IRCCS Regina Elena National Cancer Institute of Rome, Italy [RS1448/20 (2440)], and by the IRBs of each participating centre. All procedures performed were in accordance with the Helsinki Declaration. All the patients who were alive at the time of the study approval signed a specifically conceived informed consent form. For those who had deceased at the time of the analysis, a substitutive declaration of consent was obtained from their relatives.

### 2.2 Patients’ selection and data collection

Data on demographics, clinical, histopathological and immunohistochemical characteristics, treatments and related outcomes from patients’ medical records and pathology registry were retrieved. Data were anonymised, and entered into a specifically conceived database.

All the patients included were Caucasian, aged at least 18 years, had been diagnosed with triple negative breast cancer treated with standard neoadjuvant chemotherapy including anthracyclines and/or taxanes, and/or platinum derivatives, and had triple negative invasive residual disease at surgery (breast and/or axillary nodes). Post surgical adjuvant radiotherapy was delivered if indicated, and administered in all but 5 patients before capecitabine starting.

In details, pathologic assessment was performed in surgical specimens of primary tumor at definite surgery by dedicated pathologists at each participating centre as per national standards. Estrogen (ER) and progesterone receptor (PgR) status was evaluated at each centre by immunohystochemistry (IHC) according to local standard, with a cut off of ≥1% considered as positive (13). HER2 testing was performed according to the current ASCO/CAP guidelines on HER2 testing (14). An IHC score of 3+ or positive fluorescence on *in situ* hybridization or cromogenic/silver *in situ* hybridization was required to define positive HER2 status. In the *CaRe* study, triple negative tumors were defined by the absence of both hormonal receptors and of HER2 expression/amplification at definitive surgery. The investigators discussed and agreed on the inclusion of patients with a ER or PgR expression ≤ 5%. This was based on the assumption that in the case of such a low hormone receptor expression, these cancers would have clinically behaved as aggressively as in the case ER/PgR <1%. Ki67 assessment was performed in both the primary tumor and residual disease, but only this latter is reported among the descriptive characteristics of relevance. A Ki67 20% cut off was uniformly applied by all the participating centres.

All the patients included in the analysis had performed a complete restaging including contrast-enhanced total body computed tomography (CT) scan and/or 18-FDG positron emission tomography (PET) prior to capecitabine administration.

The starting dose of capecitabine was 1,250 mg/m2 twice daily from 1-14 every 21 days for 6-8 cycles. Recruited patients received treatment between January 2017 through June 2021. At least 6 cycles of adjuvant capecitabine were considered as an adequate treatment.

Treatment was administered until progression of disease, unacceptable toxicity, clinician decision or patient refusal. Median follow-up was calculated starting from the first day of capecitabine administration to the date of recurrence, death or last follow-up in all the patients recruited and in patients evaluable for effectiveness according to the study protocol.

### 2.3 Statistical methods

We retrospectively identified triple negative early breast cancer patients who received adjuvant capecitabine in clinical practice for residual disease following neoadjuvant therapy. All the aforementioned inclusion criteria had to be met.

The primary objective of the study was to evaluate the tolerability of adjuvant capecitabine in a subset of Caucasian patients having received at least one capecitabine cycle, in real-world setting. To this aim, we focused on the causes of treatment discontinuation, which included toxicity. Further reasons of treatment cessation, a part from disease progression/death, were patients’ refusal, and loss to follow up. For those patients who had discontinued capecitabine due to toxicity, we also had data on the type of toxicity reported. Available data did not include the grade of toxicity by Common Terminology Criteria for Adverse Events (CTCAE).

The secondary aim was to evaluate the effectiveness of adjuvant capecitabine in terms of DFS in the overall study population (number (N) 270 patients), and in a subset of patients with a follow-up of at least two years (N: 129) and/or for whom recurrence/death occurred within 24 months from capecitabine treatment.

Estimates of 2yr and 3yr OS were also considered of interest and included among the results presented and discussed.

Descriptive statistics were used to describe patients’ characteristics. Continuous variables were presented as medians and ranges, while categorical variables were rendered as numbers and percentages. Time-to-event endpoint definitions fully adhered to the updated standardized definition for efficacy endpoints (STEEP) in adjuvant breast cancer clinical trials. As such, DFS was evaluated from the start of capecitabine treatment to disease relapse, defined as contralateral breast cancer, local recurrence, and/or distant recurrence, and/or death from any cause. Data from patients without documented events were censored at the date of the last follow-up. Overall survival was evaluated from the start of treatment with capecitabine to death or date at last follow up (15). Survival estimates were obtained using the Kaplan-Meier product-limit method, and compared across groups using the log-rank test. Significance was defined at a p ≤ 0.05 level. Key patient- and disease-related characteristics were tested for their impact on the outcomes of interest in univariate analyses. Variables testing significant in univariate models were included in multivariate proportional hazard models developed using stepwise regression (forward selection, enter limit and remove limit, p = 0.10 and p = 0.15, respectively). Uni- and multivariate analyses were performed both in the overall study population and subset including 129 patients in reference to DFS. The assessment of interactions between significant investigational variables was taken into account when developing the multivariate model. The low number of deaths recorded at the time of data cut off made it not feasible developing inferential statistics for OS. The SPSS software (SPSS version 21.0, SPSS Inc., Chicago, Illinois, USA) was used for all statistical evaluations.

## 3 Results

From January 2017 through June 2021, 270 patients meeting the inclusion criteria and having received at least one cycle of adjuvant capecitabine were retrospectively identified. Data cutoff was set in October 2021.

Previous neoadjuvant treatments, type of definite surgery and residual disease, baseline main patient and tumor characteristics prior to capecitabine administration are reported in **Table 1**. Median age was 52 years (range, 26-77), with 124 (45.9%) patients being premenopausal at their first diagnosis. All patients were Caucasian. Neoadjuvant treatment had been delivered to all the patients. It consisted of an antracycline-based alone, taxane-based alone, platinum-taxanes based, or a combination/sequence of anthracyclines/taxanes in 1 (0.4%), 16 (5.9%), 11 (4.1%), and 242 (89.6%) patients, respectively. The median number of neoadjuvant cycles delivered was 8 (range, 2-8). One patient (0.4%) had received 2 cycles, and another patient (0.4%) 3 cycles. In 35 (13.0%) patients, the number of cycles administered had been between 4 and 8, whereas 233 (86.3%) patients had received 8 cycles. The type of surgery was conservative (quadrantectomy/lumpectomy) in 128 (47.4%) patients, while mastectomy was performed in 142 (52.6%) patients. Sentinel node biopsy was carried out in 105 (38.9%) patients, axillary dissection in 164 (60.7%) patients. One (0.4%) patient did not receive any axillary surgery due to refusal. At post-surgical assessment, 231 (85.6%) patients had infiltrating ductal carcinoma, whereas other histologies were observed in 38 (14.1%) patients, in 1 (0.4%) patient histology was unknown. Overall, 136 (50.4%) patients had residual node positive disease, 21 (7.8%) patients had large residual tumor (ypT3/ypT4) in the breast. Eighty-nine (33%) patients had very small residual disease (ypT1) without axillary involvement (ypN0). Overall, the majority of residual cancers were of grade (G) 3 (221, 81.9%), and with Ki67>20% (217, 80.4%).

**Table 1:** Characteristics of the study participants at capecitabine starting (N:270).

Characteristics	N (%)
Age in years, median (range)	52 (26-77)
Menopausal status	
*Pre-menopausal*	124 (45.9)
*Post-menopausal*	146 (54.1)
Race	
*Caucasian*	270 (100)
Performance Status_ECOG	
0	259 (95.9)
1	11 (4.1)
Number of neoadjuvant cycles, median (range)	8 (2-8)
Neoadjuvant treatment	
*Anthracycline-based alone*	1 (0.4)
*Taxane-based alone*	16 (5.9)
*Combination/sequence of anthracyclines/taxanes*	242 (89.6)
*Platinum-taxanes based*	11 (4.1)
Type of breast surgery	
*Quadrantectomy/lumpectomy*	128 (47.4)
*Mastectomy*	142 (52.6)
Type of node surgery	
*Sentinel node biopsy*	105 (38.9)
*Axillary dissection*	164 (60.7)
*None*	1 (0.4)
Infiltrating residual disease	
*Ductal carcinoma*	231 (85.6)
*Lobular carcinoma*	4 (1.5)
*Ducto-lobular carcinoma*	3 (1.1)
*Other*	31 (11.5)
*Unknown*	1 (0.4)
Estrogen/Progesterone Receptor	
*<1%*	263 (97.4)
*1-5%*	7 (2.6)
Ki67	
*≤20*	50 (18.5)
*>20*	217 (80.4)
*Unknown*	3 (1.1)
Grading	
*G1*	1 (0.4)
*G2*	43 (15.9)
*G3*	221 (81.9)
*Unknown*	5 (1.9)
Breast residual	
*No residual disease*	17 (6.3)
*ypT1/2*	229 (84.8)
*ypT3/4*	21 (7.8)
*Unknown*	3 (1.1)
Lymph node residual	
*Yes*	136 (50.4)
*No*	133 (49.3)
*Unknown*	1 (0.4)

N, number.

### 3.1 Treatment

Data on adjuvant treatments are reported in **Table 2**.

**Table 2:** Treatments administered in the adjuvant setting (N:270).

	Median (range)
**Capecitabine cycles**	6 (1-8)
	**N (%)**
Radiotherapy	
*Yes*	192 (71.1)
*No*	53 (19.6)
*Unknow*n	25 (9.3)
Endocrine therapy*	
*Yes*	7 (2.6.)
*No*	198 (73.3)
*Unknown*	65 (24.1)

*Administered in patients who had residual disease with a very low expression of only one hormonal receptor.

N, number.

Overall, adjuvant radiotherapy was administred to 192 patients, and it was delivered before capecitabine treatment in 187 (97.4%) patients, whereas it was given concomitantly to capecitabine in 5 (2.6%) patients. Seven patients (2.6%) had residual disease with a very low expression (≤5%) of only one hormonal receptor and were treated with endocrine therapy concomitantly to capecitabine. Capecitabine treatment started within 4 months from definite surgery in all of the patients.

The median number of capecitabine cycles administered was 6 (range, 1-8), with 203 (75.2%) patients having received at least 6 cycles. Sixty-seven (24.8%) patients had received less than 6 cycles but in 23 (8.5%) of them treatment was still ongoing at the time of analysis. Eighty-seven patients (32.3%) have received 8 cycles of capecitabine. Thirthy-four patients (12.6%) discontinued capecitabine treatment due to disease relapse.

### 3.2 Tolerability

Data on toxicity and treatment discontinuation are reported in **Table 3**.

**Table 3:** Reasons for capecitabine discontinuation (N:270).

Treatment discontinuation	N (%)	Type of toxicity	N (%)
*Toxicity* *Refusal/Loss to follow up* *Progressive Disease* *Total*	28 (10.4) 12 (4.4) 34 (12.6) 74 (27.4)	*HFS* *Diarrhea*, *Neutropenia* *Recurrent Mucositis* *Nausea and Vomiting* *Other toxicities* *Multiple toxic effects*	5 (1.85) 5 (1.85) 5 (1.85) 3 (1.11) 2 (0.74) 6 (2.22) 2 (0.74)

N, number; HFS, hand-foot syndrome.

Overall, among the 270 patients, 28 (10.4%) discontinued capecitabine treatment because of toxicity. Moreover, 12 (4.4%) patients refused continuing capecitabine or were lost to follow-up. Thus, if excluding discontinuation due to disease progression, 40 patients (14.8%) did not complete the planned treatment.

The toxicities which most frequently determined capecitabine discontinuation were dermatological toxicity (hand-foot syndrome), diarrhea, and neutropenia, in 5 (1.85%) per each of the toxicities listed. Treatment discontinuation due to recurrent mucositis was observed in 3 patients (1.11%), nausea and vomiting in 2 (0.74%) patients, other toxicities in 6 (2.22%) patients. Two patients (0.74%) discontinued capecitabine for multiple toxic effects.

### 3.3 Effectiveness

Overall, among the 270 patients included, 81 developed disease recurrence (30%). Thirty-four (42%) patients recurred while on treatment (23 before the sixth cycle and 11 after the sixth cycle of capecitabine) and 47 (58%) recurred after the end of adjuvant capecitabine.

In 13 (16%) patients, a local recurrence was observed; in one (1.2%) patient, we observed contralateral breast cancer; in 52 (64.2%) patients, disease progression was characterized by the development of distant metastases; in 15 patients (18.5%), disease progression occurred at multiple sites. The site of distant metastases most frequently observed was viscera and nodes, whereas bone-only metastases were observed in 11 patients, and only 2 cases of brain metastases were observed so far.

**Figure 1:**
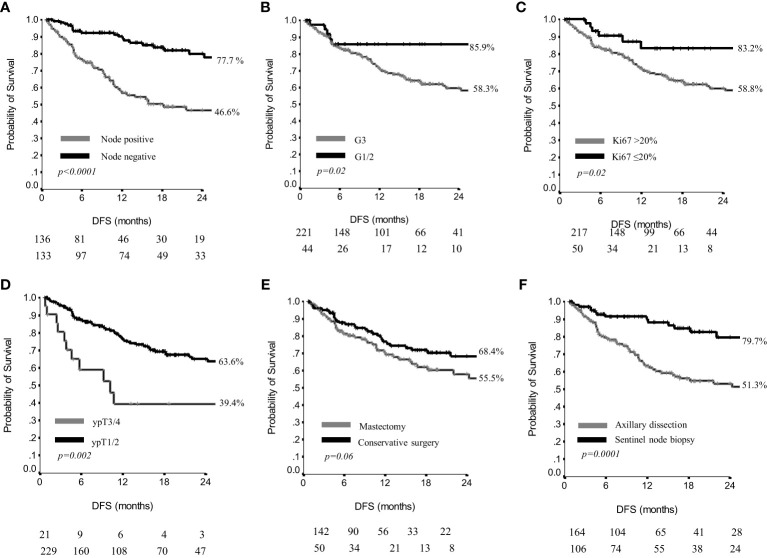
Disease free survival by nodal status **(A)**, grading **(B)**, Ki67 **(C)**, residual breast disease **(D)**, breast surgery **(E)** and axillary surgery **(F)** in 270 patients.

When considering the entire study population (N: 270), at a median follow-up of 15 months (range 1-54), we observed a 2yr DFS of 62%, with a median DFS still not reached (**Supplementary Figure 1**). The most unfavorable results were observed in patients with residual axillary positive nodes, being the 2yr DFS 46.6%, and the median DFS 18 months. In the node negative subgroup, the 2yr DFS was 77.7% and the median DFS is not reached (p<0.0001**)** (**Figure 1A**). Residual G1/G2 tumors had a 2yr DFS of 85.9%, and the median DFS was not reached; conversely, G3 tumors had 2yr DFS of 58.3% with a median DFS of 32 months (p=0.02) (**Figure 1B**). Residual tumors with Ki67 ≤ 20% showed a 2yr DFS of 83.2%, and a median DFS not reached, whereas tumors with Ki67 >20% had a 2yr DFS of 58.8% and a median DFS of 32 months (p=0.02) (**Figure 1C**). A small residual in the breast was related to more favorable DFS results, since ypT1/ypT2 versus ypT3/ypT4 showed a 2yr DFS of 63.6% with a median DFS not reached, versus a 2yr DFS of 39.4% and a median DFS of 10 months (p=0.002) (**Figure 1D**).

The type of breast surgery was not significantly related to the DFS outcome (p=0.06) (**Figure 1E**). Conversely, patients having received a sentinel node biopsy had a 2yr DFS of 79.7% and the median DFS was not reached, whereas patients treated with axillary dissection had a 2yr DFS of 51.3%, with a median DFS of 28 months (p=0.0001) (**Figure 1F**).

**Figure 2:**
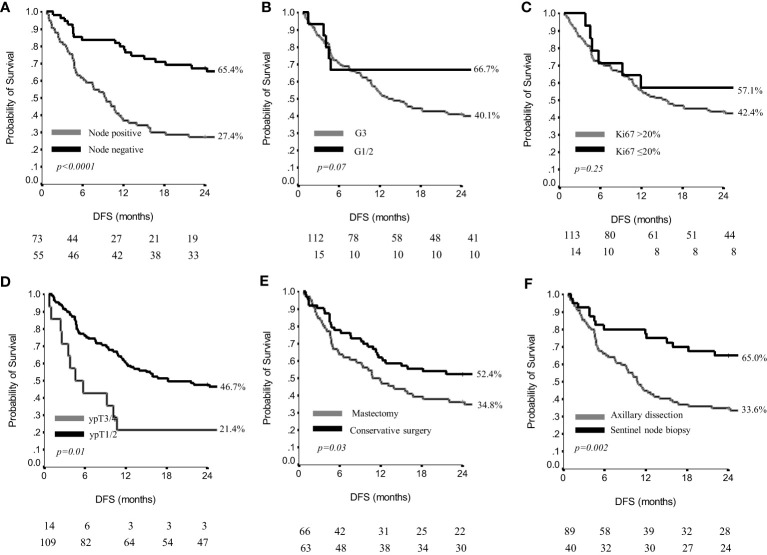
Disease free survival by nodal status **(A)**, grading **(B)**, Ki67 **(C)**, residual breast disease **(D)**, breast surgery **(E)** and axillary surgery **(F)** in 129 patients.

By study protocol, 129 patients were evaluable for effectiveness, since they had a minimum of 24 months of follow-up or disease recurrence within 24 months (72 patients, 55.8%). At a median follow-up of 25 months (range, 2-54), we observed a 2yr DFS of 43.4%, and a median DFS of 16 months (**Supplementary Figure 2**). The most unfavorable results were observed in patients with residual axillary positive nodes, being the 2yr DFS 27.4%, and the median DFS 9 months. In the node negative subgroup, the 2yr DFS was 65.4% and the median DFS is not reached (p<0.0001**)** (**Figure 2A**). Conversely, grading and Ki67 did not affect DFS results (**Figures 2B, C**). A small residual in the breast was related to more favorable DFS outcomes, since ypT1/ypT2 versus ypT3/ypT4 showed a 2yr DFS of 46.7% with a median DFS of 18 months, versus a 2yr DFS of 21.4% and a median DFS of 5 months (p=0.01) (**Figure 2D**). Type of surgery was related to DFS results. In more detail, patients having undergone a conservative breast surgery showed a 2yr DFS of 52.4% and the median DFS was not reached, whereas patients having undergone mastectomy showed a 2yr DFS of 34.8% with a median DFS of 11 months (p=0.03) (**Figure 2E**). Similarly, patients having received a sentinel node biopsy had a 2yr DFS of 65.0% and the median DFS was not reached, whereas patients treated with axillary dissection had a 2yr DFS of 33.6% with a median DFS of 11 months (p=0.002) (**Figure 2F**).

Detailed results on DFS are reported also in **Table 4**.

**Table 4:** Disease free survival in the overall study population (N:270) and in patients with a minimum of 24-month follow-up (N:129).

	2yr DFS (%) (SE)	mDFS (95% CI)	p value	HR (95% CI)
	**N:270**			
	62.0 (0.04)	NR		
**Nodal status** *Positive* *Negative*	46.6 (0.06) 77.7 (0.05)	18 (3-33) NR	<0.0001	3.127 (1.935-5.054)
**Size of residual breast disease** *ypT3/4* *ypT1/2*	39.4 (0.12) 63.6 (0.04)	10 (4-16) NR	0.002	2.623 (1.383-8.976)
**Grading** *3* *1/2*	58.3 (0.04) 85.9 (0.06)	32 (27-38) NR	0.02	2.720 (1.100-6.727)
**Ki67** *>20* *≤20*	58.8 (0.04) 83.2 (0.07)	32 (26-38) NR	0.02	2.646 (1.150-6.091)
**Breast surgery** *Mastectomy* *Quadrantectomy/lumpectomy*	55.5 (0.06) 68.4 (0.05)	32 (21-43) NR	0.06	1.518 (0.973-2.367)
**Axillary surgery** *Axillary dissection* *Sentinel node biopsy*	51.3 (0.05) 79.7 (0.05)	28 (15-40) NR	0.0001	2.813 (1.647-4.804)
	**N:129**			
	43.4 (0.04)	16 (7-24)		
**Nodal status** *Positive* *Negative*	27.4 (0.05) 65.4 (0.06)	9 (6-12) NR	<0.0001	2.788 (1.720-4.519)
**Size of residual breast disease** *ypT3/4* *ypT1/2*	21.4 (0.11) 46.7 (0.05)	5 (1-8) 18 (7-29)	0.01	2.283 (1.201-4.340)
**Grading** *3* *1/2*	40.1 (0.05) 66.7 (0.12)	13 (9-18) NR	0.07	2.279 (0.921-5.639)
**Ki67** *>20* *≤20*	42.4 (0.05) 57.1 (0.13)	16 (8-24) NR	0.25	1.627 (0.708-3.741)
**Breast surgery** *Mastectomy* *Quadrantectomy/lumpectomy*	34.8 (0.06) 52.4 (0.06)	11 (6-15) NR	0.03	1.647 (1.056-2.567)
**Axillary surgery** *Axillary dissection* *Sentinel node biopsy*	33.6 (0.05) 65.0 (0.08)	11 (8-13) NR	0.002	2.300 (1.344-3.936)

N, number; yr: year; SE, standard error; mDFS, median disease free survival (months); CI, confidence interval; HR, hazard ratio; NR, not reached.

**Figure 3:**
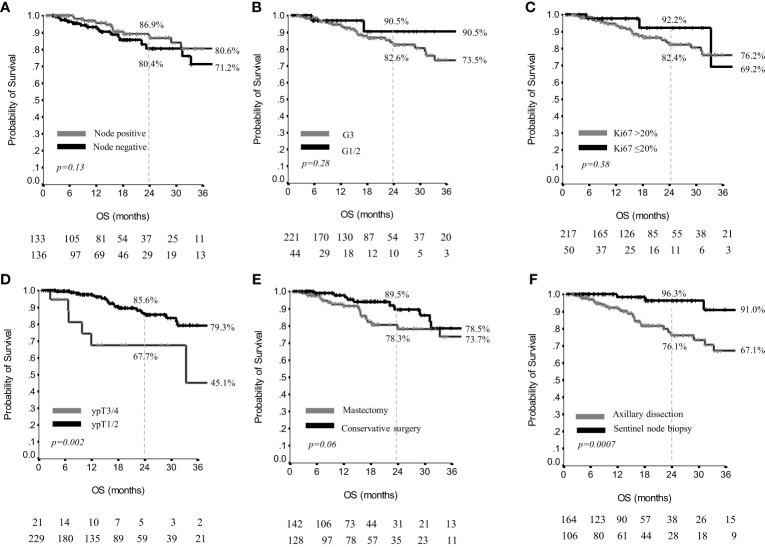
Overall survival by nodal status **(A)**, grading **(B)**, Ki67 **(C)**, residual breast disease **(D)**, breast surgery **(E)** and axillary surgery **(F)** in 270 patients.

Results on OS are reported in **Table 5**. In the overall patients’ population, the 2yr and 3yr OS were 84.0% and 76.2%, respectively, and the median OS was not reached (**Supplementary Figure 3**). Definite axillary nodal status, grading and Ki67 did not significantly affect OS results (**Figures 3A–C**). Conversely, the size of residual tumor in the breast influenced OS, since 2yr and 3yr OS were 85.6% and 79.3% in ypT1/pT2, versus 67.7% and 45.1% in ypT3/pT4 (p=0.002), (**Figure 3D**).

**Table 5:** Overall survival in the overall study population (N:270) and in patients with a minimum of 24-month follow-up (N:129).

	2yr OS (%) (SE)	3yr OS (%) (SE)	p value	HR (95% CI)
	**N:270**			
	84.0 (0.03)	76.2 (0.05)		
**Nodal status** *Positive* *Negative*	86.9 (0.04) 80.4 (0.05)	80.6 (0.06) 71.2 (0.08)	0.13	1.737 (0.835-3.611)
**Size of residual breast disease** *ypT3/4* *ypT1/2*	67.7 (0.12) 85.6 (0.03)	45.1 (0.20) 79.3 (0.05)	0.002	3.886 (1.566-9.644)
**Grading** *3* *1/2*	82.6 (0.04) 90.5 (0.07)	73.5 (0.05) 90.5 (0.07)	0.28	2.173 (0.517-9.126)
**Ki67** *>20* *≤20*	82.4 (0.04) 92.2 (0.06)	76.2 (0.05) 69.2 (0.20)	0.38	1.696 (0.513-5.604)
**Breast surgery** *Mastectomy* *Quadrantectomy/lumpectomy*	78.3 (0.05) 89.5 (0.04)	73.7 (0.07) 78.5 (0.07)	0.06	2.058 (0.961-4.406)
**Axillary surgery** *Axillary dissection* *Sentinel node biopsy*	76.1 (0.05) 96.3 (0.03)	67.1 (0.07) 91.0 (0.06)	0.0007	6.150 (1.864-20.294)
	**N:129**			
	78.0 (0.04)	70.8 (0.05)		
**Nodal status** *Positive* *Negative*	75.0 (0.06) 81.0 (0.05)	66.4 (0.08) 75.1 (0.06)	0.24	1.541 (0.740-3.209)
**Size of residual breast disease** *ypT3/4* *ypT1/2*	58.7 (0.14) 81.4 (0.04)	39.2 (0.19) 75.3 (0.05)	0.005	3.420 (1.378-8.490)
**Grading** *3* *1/2*	76.6 (0.04) 84.9 (0.10)	68.2 (0.06) 84.9 (0.10)	0.35	1.943 (0.463-8-161)
**Ki67** *>20* *≤20*	77.0 (0.04) 85.1 (0.10)	71.2 (0.05) 63.8 (0.20)	0.69	1.277 (0.387-4.276)
**Breast surgery** *Mastectomy* *Quadrantectomy/lumpectomy*	69.7 (0.06) 86.6 (0.05)	65.6 (0.07) 75.9 (0.07)	0.05	2.090 (0.977-4.472)
**Axillary surgery** *Axillary dissection* *Sentinel node biopsy*	69.6 (0.06) 94.4 (0.04)	61.4 (0.07) 89.2 (0.06)	0.002	5.295 (1.604-17.483)

N, number; yr, year; OS: overall survival; SE, standard error; HR, hazard ratio; CI, confidence interval.

Type of breast surgery did not significantly affect 2 and 3yr OS (p=0.06) (**Figure 3E**), whereas in patients treated with sentinel node biopsy, the 2yr and 3yr OS were 96.3% and 91.0%, versus 76.1% and 67.1% in patients having received axillary dissection (p=0.0007) (**Figure 3F**).

**Figure 4:**
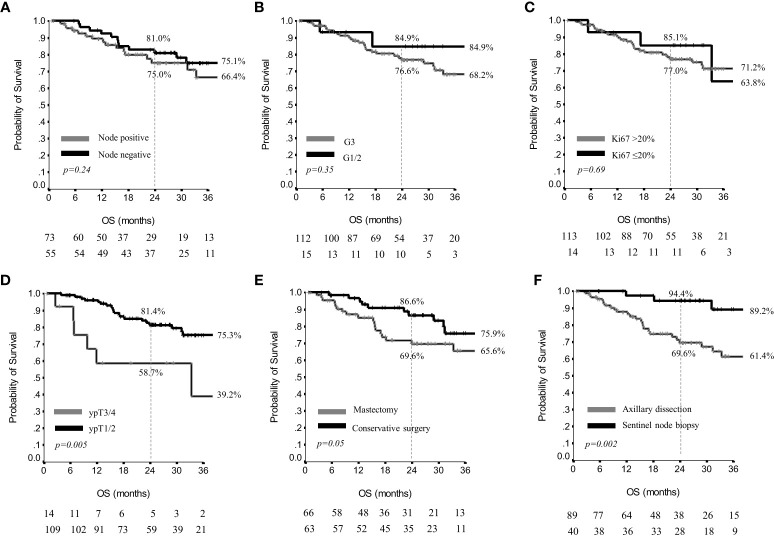
Overall survival by nodal status **(A)**, grading **(B)**, Ki67 **(C)**, residual breast disease **(D)**, breast surgery **(E)** and axillary surgery **(F)** in 129 patients.

In the subset of 129 patients with a minimum 24 months follow-up, who also included 23 patients who had died within 24 months from the start of adjuvant capecitabine, the 2yr OS was 78.0%, and the 3yr OS was 70.8%, while the median OS values were not reached (**Supplementary Figure 4**). As concerns tumor residual characteristics and OS results, definite axillary nodal status, G and Ki67 did not significantly affect OS results (**Figures 4A–C**). Conversely, the size of residual tumor in the breast influenced OS, since 2yr and 3yr OS were 81.4% and 75.3% in ypT1/ypT2, versus 58.7% and 39.2% in ypT3/ypT4 (p=0.005) (**Figure 4D**). Two and 3yr OS were 86.6% and 75.9% in patients treated with conservative breast surgery, versus 69.7% and 65.6% in patients having received mastectomy (p=0.05) (**Figure 4E**). Moreover, in patients treated with sentinel node biopsy the 2yr and 3yr OS were 94.4% and 89.2%, versus 69.6% and 61.4% in patients having received axillary dissection (p=0.002) (**Figure 4F**).

Multivariate analysis for DFS were also conducted in the overall study population (N: 270) and in pre-defined subset of 129 patients.

In univariate analysis including data from the overall study population, axillary dissection, large size residual tumors, node positivity, G3, and Ki67>20% tumors were associated with a significantly shorter DFS. In multivariate analysis, these associations were only partially confirmed. Patients with positive nodes at definite surgery had a significantly worse DFS (HR=3.380, p<0.0001) compared to their counterpart. Similarly, patients with G3 residual tumors had worse DFS (HR= 3.395, p=0.011) than patients with G1/G2 tumors, and patients with small tumor size had more favorable outcome compared to the greater tumour sizes (HR 0.450, p=0.021 for ypT1/2 vs ypT3/4) (**Table 6**). In multivariate analyses including data from the subset of 129 patients, nodal status was the only factor impacting DFS significantly (HR 3.083, p <0.0001) (**Table 7**).

**Table 6:** Uni- and multivariate analysis of disease free survival in the overall study population (N: 270).

DFS	Univariate Analysis		Multivariate Analysis	
	HR (95% CI)	p value	HR (95% CI)	p value
Breast surgery				
*Mastectomy vs Quadrantectomy/lumpectomy*	1.518 (0.973-2.367)	0.066	**-**	ns
Axillary surgery				
*Axillary dissection vs* *sentinel node biopsy*	**2.813 (1.647-4.804)**	**<0.0001**	–	ns
Size of residual breast disease		0.013		0.024
*ypT12 vs ypT0*	1.035 (0.325-3.297)	0.953	2.405 (0.576-10.051)	0.229
*ypT34 vs ypT0*	2.692 (0.750-9.661)	0.129	**5.319 (1.176-24.333)**	**0.030**
*ypT12 vs ypT34*	**0.385 (0.203-0.729)**	**0.003**	**0.450 (0.228-0.886)**	**0.021**
Nodal status				
*positive vs negative*	**3.127 (1.935-5.054)**	**<0.0001**	**3.380 (2.051-5.570)**	**<0.0001**
Grading				
*G3 vs G1/2*	**2.720 (1.100-6.727)**	**0.030**	**3.395 (1.328-8.678)**	**0.011**
Ki67				
*>20 vs ≤20*	**2.646 (1.150-6.091)**	**0.022**	0.993 (0.988-0.999)	0.084

DFS, disease free survival; N, number; HR, hazard ratio; CI, confidence interval, ns, not significative. We report in bold those values that reached the statistical significance (the treshold for statistical significance is reported in the Methods).

**Table 7:** Uni- and multivariate analysis of disease free survival in patients with a minimum 24-month follow-up (N: 129).

DFS	Univariate Analysis		Multivariate Analysis	
	HR (95% CI)	p value	HR (95% CI)	p value
Breast surgery				
*Mastectomy vs Quadrantectomy/lumpectomy*	**1.647 (1.056-2.567)**	**0.028**	1.498 (0.951-2.360)	0.081
Axillary surgery				
*Axillary dissection vs* *sentinel node biopsy*	**2.300 (1.344-3.936)**	**0.002**	–	ns
Size of residual breast disease	-	0.045		
*ypT12 vs ypT0*	0.730 (0.229-2.327)	0.594	**-**	ns
*ypT34 vs ypT0*	1.639 (0.455-5.906)	0.450		
*ypT12 vs ypT34*	**0.445 (0.234-0.846)**	**0.014**		
Nodal status				
*positive vs negative*	**2.788 (1.720-4.519)**	**<0.0001**	**3.083 (1.889-5.031)**	**<0.0001**
Grading				
*G3 vs G1/2*	2.279 (0.921-5.639)	0.075	**-**	ns
Ki67				
*>20 vs ≤20*	1.627 (0.708-3.741)	0.252	**-**	ns

DFS, disease free survival; N, number; HR, hazard ratio; CI, confidence interval; ns, not significative. We report in bold those values that reached the statistical significance (the treshold for statistical significance is reported in the Methods).

Data analysis by number of capecitabine cycles delivered in the whole patients’ population, showed a median DFS of 3.1 (95%CI,1.9-4.3) months in patients having received less than 6 cycles, whereas it was 18.2 (95%CI,5.4-31.0) months in patients having received at least 6 cycles (p=0.002). Moreover, the 2yr DFS was 28.7% and 48.0% in the two groups, respectively (p=0.002) (**Supplementary Figure 5**). The median OS was not reached. Two and 3yr OS were both 59.6% in patients treated with less than 6 capecitabine cycles, whereas it was 83.0% and 73.7% in patients treated with at least 6 cycles (p=0.03) (**Supplementary Figure 6**). These statistically relevant differences in DFS and OS no longer existed when excluding patients whose disease had progressed during capecitabine treatment, namely the 23 patients who had progressed before the sixth cycle and the 11 patients who had relapsed after the sixth cycle.

In the whole patients’ population, we also examined data on patients having received 6 or 8 cycle of capecitabine. Overall, data on the 98 patients having received 6 cycles were as follow: median DFS was 34 months (95%CI,27-41), the 2yr DFS was 63.0%. The median OS was not reached, and the 2yr and 3yr OS were 88.8% and 84.6%, respectively. Among the 87 patients having received 8 cycles of capecitabine, the median DFS was not reached, and the 2yr DFS was 65.8%. The median OS was 46 months (95%CI,2-69) and the 2yr and 3yr OS were 82.8% and 65.8%, respectively. Differences between the two groups were not statistically significant, being p values =0.29 and 0.32, for DFS and OS, respectively.

In 55 patients having received 6 cycles of capecitabine and having a follow-up of at least 24 months, the 2yr DFS was 47.3%, the median DFS was 18 months (95%CI,4-33), whereas the 2yr and 3yr OS were 85.1% and 81.0% with a median OS not reached. When analyzing 34 patients having received 8 capecitabine cycles and having a follow-up of at least 24 months, the 2yr DFS was 44.1%, the median DFS was 14 months (95%CI,1-27), whereas the 2yr and 3yr OS were 78.3% and 62.3%, with a median OS of 46 months (95%CI,23-69). The difference between the above reported two groups were not statistically significant, being p values =0.76 and 0.26, for DFS and OS, respectively.

## 4 Discussion

We herein present results from the *CaRe* study, a multicenter, observational, retrospective study of adjuvant capecitabine in triple negative early breast cancer patients with invasive residual disease at surgery following neoadjuvant chemotherapy. The efforts of 44 Italian collaborating Institutions coordinated by the IRCCS Regina Elena National Cancer Institute of Rome, Italy, allowed the collection and analysis of tolerability and effectiveness data related to 270 patients meeting the eligibility criteria. We primarily aimed to address tolerability, reported in descriptive analysis on the potential causes of treatment discontinuation, along with refusal to treatment continuation and loss to follow-up. Our secondary aim was effectiveness. We presented data on 2yr DFS and median DFS, and 2 and 3yr OS. Data were analyzed in the overall study population (N: 270) and in a subset of patients with a minimum follow-up length of 24 months or whose disease had progressed within 24 months (N: 129).

Evidence from the *CaRe* study supports an unexpectedly low toxicity of adjuvant capecitabine in Caucasian patients. The paucity of inherent real world evidence, mostly coming from advanced setting, invites extreme caution in formulating any supporting hypotheses. We may only suppose that the compliance to the treatment administered, possibly influenced by the positive efficacy results of the *CREATE-X* study, and the better general conditions of patients from adjuvant setting may have positively influenced our results on the particularly low discontinuation rate of adjuvant capecitabine.

To the aim of further discussion, the *CREATE-X* (9) represents the most suitable term of comparison for our results. Bearing this in mind, underlying the relevant differences between this latter trial and our study will guide and inform the comparison in results across these two studies. As previously mentioned, the *CREATE-X* exclusively enrolled patients from Japanese and Korean Institutions, while the entirety of our population is Caucasian. Pharmacokinetics and pharmacodinamic issues may thus limit the generalizability of the findings from the *CREATE-X* to our work. Secondarily, the subgroup of patients diagnosed with triple negative breast cancer accounted for about the 32% of the *CREATE-X* population, i.e., 286 patients, of whom 139 were randomly assigned to capecitabine. This latter subgroup is significantly smaller in size compared to our study population (N: 270), which is solely represented by triple negative breast cancer patients. However, in our study, data concerning outcomes extend to a minimum of 24 months exclusively for 129 patients, with the median follow-up for the overall population being of 15 months. The immaturity of our efficacy outcomes imposes caution in results interpretation. A subsequent effectiveness analysis of the *CaRe* study has been planned at a median follow-up of 24 months for the overall study population (N: 270 patients).

A further relevant issue is related to the different study designs, i.e., a randomized clinical trial vs an observational study carried out according to a retrospective approach. It is also noteworthy and largely dipendent on the retrospective nature of our study that, when addressing the tolerability issues, we exclusively reported on the causes of treatment discontinuation. Given the aforementioned distinctive traits, in the *CaRe* study, capecitabine discontinuation due to toxicity was only observed in the 10.4% of our patients. These data support an unexpectedly good treatment tolerability. Even when considering patients who refused treament continuation or were lost to follow-up, the rate of capecitabine discontinuation not related to disease progression was 14.8%, lower than that reported by the authors of the *CREATE-X*. In the *CaRe* study, no new safety issues emerged, with dermatological, gastrointestinal toxicities and myelosuppression being the most frequently observed side effects having caused treatment discontinuation. Overall, the good tolerability observed in the *CaRe* patients, all Caucasians, is a relevant and awaited information concerning non Asiatic patients. The effectiveness of adjuvant capecitabine in the present study appears to be lower than the efficacy reported in the *CREATE-X*. Indeed, in the 270 patients with a median follow-up of 15 months, which still remains particularly short, we observed a 2yr DFS of 62%, whereas in the *CREATE-X* the 5yr DFS was 69.8% in the triple negative subgroup. Also the 2yr and 3yr OS in the *CaRe* study were 84.0% and 76.2%, whereas the 3yr and 5yr OS in the *CREATE-X* were 94.0% and 89.2%, but data reported are on the whole patients’ population, not specifically in the triple negative subset. The 5yr OS in the triple negative subgroup in the *CREATE-X* was 78.8%. In the *CaRe* study, survival estimates for capecitabine evaluated in 129 patients having a minimum follow-up of 24 months confirmed lower effectiveness compared to the *CREATE-X*. The unfavorable outcome in the *CaRe* population may at least partly be explained by the disease characteristics emerged at the pathological assessment of surgical specimens. Indeed, 136 patients showed residual node positive disease (50.4%) and overall, the majority of residual disease was G3 (81.9%), and with Ki67 >20% (80.4%). Patients who received a major surgery (mastectomy and/or axillary dissection) showed a worse outcome, but this is presumably related to a surgery choice based on initial tumor characteristics, where more advanced tumors underwent a more radical surgery. Unfortunately, baseline triple negative subgroup characteristics and type of surgery were not made available by the authors of the *CREATE-X.* Differences in the number of capecitabine cycles may deserve further mentioning. In the *CREATE-X*, the 64.11% of the patients received 8 cycles of capecitabine, against the 32.3% in the *CaRe* study. Differences between the study designs do not allow direct comparison, since in the *CaRe s*tudy a number of 6-8 cycles was allowed, whereas in the *CREATE-X* the established number was 8, following an amendement after the first 50 patients treated with 6 cycles. However, when comparing data in patients having received 6 and 8 cycles, no significant differences emerged from our data.

Evidence on the use of adjuvant or extended treatment with capecitabine in 876 patients with operable triple negative breast cancer patients after neo/adjuvant treatment with antracyclines and/or taxane chemotherapy has recently come from the *GEICAM/2003-11-CIBOMA/2004-01* study (16). Overall, the study failed to show a benefit in DFS of adding capecitabine in patients with early triple negative breast cancer. At a median follow-up of 7.3 years, capecitabine extended/adjuvant treatment did not prolong DFS with respect to the observational arm (HR 0.82, p=0.136). Exclusively the subgroup of patients with non-basal tumor seemed to benefit from capecitabine, with a DFS HR of 0.53 versus 0.94 in the basal subgroup (p=0.069), and an OS with a HR of 0.42 versus 1.23 in the basal subtype (p=0.005). The tolerability of capecitabine treatment was as expected, with 75.2% of the patients completing 8 cycles. In the attempt to interpret this study results, the higher toxicity related to capecitabine-in the *GEICAM/2003-11-CIBOMA/2004-01* trial compared with the *CREATE-X* may have determined a decreased relative dose-intensity possibly affecting treatment outcomes. Further evidence on the outcome of triple negative patients with residual disease greater than 1 centimeter following neoadjuvant chemotherapy stems from the *ECOG-ACRIN EA1131* trial (17). Patients were randomly assigned to adjuvant carboplatin/cisplatin for 4 cycles or capecitabine for 6 cycles. Basal versus non-basal subtype was determined by PAM50 in the residual disease at surgery. Platinum treatment did not improve outcomes in patients with basal subtype, and caused higher toxicity. This trial was closed prematurely, and the conclusions of the authors were that patients enrolled had lower than expected 3yr invasive DFS in both arms. To the aim of our discussion, the authors reported a 3yr OS of 66% in the capecitabine arm for the the basal subtype at a median follow-up of 20 months. These estimates are not dissimilar from that of the *CaRe* study. However, no evaluation of the molecular subtypes was performed in our study.

Results from the *CREATE-X* contrast with those from the *GEICAM/2003-11*, and *ECOG-ACRIN EA1131*. Substantial differences across these trials by study design and patient population may at least partly clarify inconsistency in outcomes. The *CREATE-X* was exclusively conducted in a post-neoadjuvant setting and including a relatively small subset of particularly high risk patients, i.e., triple negative breast cancer patients’ tumors with residual disease at surgery following neoadjuvant treatment. Moreover, 42% of all the patients randomized to capecitabine in the *CREATE-X* showed response to neoadjuvant treatment, compared with 60% of patients in the *ECOG-ACRIN EA1131* study. In addition, in this latter trial, residual disease was submitted for PAM50 analysis for triple negative breast cancer (TNBC) subtype determination (basal *vs* nonbasal). Diverse ethnicityies were included, i.e., 70% of participants were white, 20% black, and 10% Hispanic. Patients with basal subtype TNBC had worse invasive DFS than patients with nonbasal subtype TNBC (HR = 1.71; 95% CI, 1.10 to 2.67) (17). In reference to our study, we did not apply the PAM 50 to discern between basal and non basal TNBC and have no data concerning this specific aspect in our study population. Theoretically, a diverse percent representation may at least partly explain differences in DFS. However, even if these data were available, it is not entirely clear whether the distribution of the TNBC subgroups is fully comparable across different ethnicities and we cannot exclude that a 30% difference in the representation of the Caucasian ethnicity between the EA1131 and the CaRe may translate into a different representation of basal and non basal TNBC, which may itself translate into different treatments outcomes (17).

Finally, there is paucity of evidence concerning the use of adjuvant capecitabine in real-world setting.

Vilbert and co-authors reported on 21 patients with triple negative breast cancer treated, in clinical practice after neoadjuvant therapy, with adjuvant capecitabine at a starting dose of 1,000 mg/m2 bid, up to a maximum of 8 cycles, or observation. Only 43% of the patients completed the treatment planned. At a median follow-up of 23 months, the 3yr DFS was 76.2% and the 3yr OS was 81%. The median relapse free survival was 30.8 months in the capecitabine group, more favorable than that observed in patients not having received adjuvant treatment, which was 20 months (HR 0.23, p=0.045). Toxicity was as expected, and a dose-reduction or treatment delay were necessary in more than 60% of the patients (18). Another small case-series was carried out in US by Beyerlin and colleagues, who recruited 23 triple negative breast cancer patients with residual disease at definite surgery. At a median follow-up of 14 months, the median DFS, calculated on the whole patients’ populations, was 22 months, with 30.4% of patients having recurred. The 2yr DFS was 42.8%, lower than that reported in the whole population of the *CaRe* study. Tolerability of the treatment was poor, lower than that of the *CREATE-X*, with 34.8% of patients who discontinued the planned treatment (19).

The analysis of the *CaRe* study has some limitations. The immaturity of our outcome data on effectiveness has been previously discussed. Of further relevance, the retrospective nature and the heterogeneity of our patients’population, due to the multicentric and observational design. The lack of central pathologic assessment of residual disease in post-surgical samples represents a relevant limitation of the CaRe study. Residual volume grades were evaluated by expert pathologists operating at the single Institution level. We lack detailed information on the criteria regulating assessment and reporting of residual tumor volume at each of the participating centres. Indeed, central pathologic assessment was not envisioned by the study protocol, although this would have further increased the quality of our study results and will surely be envisioned in future studies within this research pipeline. Further weaknesses are the lack of information concerning the BRCA status, molecular subtypes, detailed data on some baseline patient and disease characteristics and on capecitabine toxicity, dose reductions and the Relative Dose Intensity. More specifically, the lack of data concerning clinically and instrumentally dectected T and N, i.e., cTN, may reduce the overall ability to compare our patients characteristics to those from other trials. However, in reference to the CREATE-X, such comparison would have anyways been halted by the lack of distinctions in reporting baseline patients and diseases’ characteristics by molecular subtype, i.e., TNBC and HER2 negative luminal cancer patients. Still, completeness of reporting on the pathologic characteristics inherent to both the residual T and N as assessed in surgical samples post neo-adjuvant therapy, somewhat counterbalances our weakness in terms on baseline cTN. In reference to capecitabine tolerability, our results would have significantly benefited by the availability of data concerning dose reductions and delays in capecitabine administration, which unfortunately we do not have.

Another potential source of selection may have been introduced by the general tendency of medical oncologists to avoid administration of adjuvant capecitabine due to lack of consistent evidence related to treatment tolerability and effectiveness. This attitude may have led to administer capecitabine exclusively to patients with particularly unfavorable prognostic factors.

Our work also has relevant strengths. In first place, to the best of our knowledge, the *CaRe* study is thus far the largest real-world experience concerning adjuvant capecitabine in Caucasian patients with triple negative residual disease after neoadjuvant treatment. This analysis provides a picture on tolerability and effectiveness of the drug in this unfavourable subset of patients, in a not selected patients’ population.

In doing so, it efficaciously integrates the data from the *CREATE-X*, a randomized clinical trial whose population by study design had to meet previously stated selection criteria, which do not always extend to the general population from the real-world setting. Along with the matters previously debated on the population ethnicity, this may concur to reduce the external generalizability of the *CREATE-X* results and make the inherent evidence poorly applicable to the population treated in general practice.

Given the poor efficacy results obtained with adjuvant capecitabine, future directions point to escalating strategies employing new drugs as post-neoadjuvant treatments, such as Poly (ADP-ribose) polymerase (PARP) (20, 21), and immune checkpoint inhibitors (22–24), both under investigation particularly in the triple negative patients’subset.

To date, no standard therapies are established for triple negative breast cancer patients with residual disease after neoadjuvant treatment. The *CREATE-X* trial, notwithstanding the limited sample size of the patients’ population, represents a milestone, being the first randomized trial showing an advantage in outcomes for this subset of patients. Other published trials reported discordant results. The *CaRe* study includes a remarkably high number of patients treated in real-world setting, and shows a low rate of toxicity-related discontinuation. It certainly represents a relevant contribution in treatment decision-making in Caucasian patients with triple negative residual disease. Our results on effectiveness seem less favorable than those from the *CREATE-X* presumably for the aforementioned reasons. Increasingly precise and innovative tools are hugerly needed to help select those patients and tumors characteristics which contribute to depict the ideal therapeutic target population, i.e., the patients’ subset which may best benefit from additional adjuvant treatments, including capecitabine.

## 5 Conclusions

The *CaRe* study was aimed to produce data on tolerability and effectiveness of adjuvant capecitabine administered in clinical practice to triple negative breast cancer patients with pathologically assessed triple negative residual disease. Our first time finding on an exceptionally low rate of treatment discontinuation due to toxicity is quite unexpected, and eagerly awaited in Caucasian patients. Outcomes data, still immature, seem to suggest low effectiveness of adjuvant capecitabine. However, our results are only partly comparable to those from the most relevant trials, i.e, the *CREATE-X*. Overall, results from the *CaRe* study, particularly in light of the inclusion of a relatively high number of patients treated in real-world setting, certainly represent a relevant contribution in informing treatment decisions in this subgroup of patients with particularly unfavorable outcomes, and encourage further research.

## Data availability statement

The datasets presented in this study can be found in online repositories. The names of the repository/repositories and accession number(s) can be found below: GARRbox repository [https://gbox.garr.it/garrbox/index.php/s/yI8JHqXpfdTVXpz].

## Ethics statement

The studies involving human participants were reviewed and approved by: Comitato Etico Centrale IRCCS – sezione IFO – Fondazione Bietti, Comitato Etico Regione Toscana - Area Vasta Centro, Comitato Etico Lazio 1, Comitato Etico della Fondazione Policlinico Universitario Agostino Gemelli IRCCS Università Cattolica del Sacro Cuore, Comitato Etico dell’Università Campus Biomedico di Roma, Comitato Etico Cardarelli-Santobono di Napoli, comitato etico ATS Sardegna, Comitato Etico Palermo 1, CER Umbria - Comitato Etico Regionale Umbria, Comitato Etico Lazio 2, comitato etico della Romagna – CEROM, Comitato Etico Interaziendale AOU Città della Salute e della Scienza di Torino, Comitato Etico Brianza, Comitato etico per le sperimentazioni cliniche dell’azienda provinciale per i servizi sanitari, Trento, Comitato Etico Regione Marche, Comitato Etico dell’Insubria, Comitato Etico Indipendente Istituto Clinico Humanitas, Comitato etico per la sperimentazione clinica (Cesc) della provincia di Treviso e Belluno, Comitato etico per la sperimentazione clinica delle provincie di Verona e Rovigo, Comitato Etico di Brescia, Comitato Etico Interaziendale ASO S.Croce e Carle e AA.SS.LL CN1, CN2 e AT, Comitato Etico dell’Università “Sapienza” Di Roma, Comitato Etico per le province dell’Aquila e Teramo, Comitato Etico delle province di Chieti e Pescara. Please note that more canters refer to one Ethics committee. The patients/participants provided their written informed consent to participate in this study. This study was conducted according to the guidelines of the Declaration of Helsinki, and reviewed and approved by the Institutional Review Board (IRB) of the coordinating center, the IRCCS Regina Elena National Cancer Institute of Rome, Italy [RS1448/20(2440)], and by the IRBs of each participating center enrolling patients. All the patients who were alive at the time of the study approval signed a specifically conceived informed consent form. For those who had deceased at the time of the analysis, a substitutive declaration of consent was obtained from their relatives.

## Author contributions

PV conceived and designed the study, analyzed and interpreted the data and drafted the manuscript. FSDL and MB analyzed and interpreted the data and drafted the manuscript. LP, EK, KC, UDG, FB, JF, ACar, AF, EP, MMit, VS, SSt, GT, DS, AG, NT, OG, GSar, LLi, IM, GD’A, MV, TG, MPi, RB, ER, SA, MEC, FZu, FCap, LLa, RT, SSc, AB, RS, IP, MMu, ACas, LG, VG, EMV, FZo, EF, MAF, MMa, EMR, RP, MRV, LF, MMi, PS, MR, AA, RDV, MPo, FR, FGr, MG, MM-S, AZ, CB, FP, SC, FCav, EV, EC, ST, IoS, made substantial contributions to the acquisition of data. IsS analyzed the data. NLV, ARG, FGi, VL, LR, AM, BF, LM, GSan, FT, PM, and GC critically revised the manuscript. All authors contributed to the article and approved the submitted version.
