# Supplementary material for: Adjuvant capecitabine in triple negative breast cancer patients with residual disease after neoadjuvant treatment: real-world evidence from CaRe, a multicentric, observational study
Source: Front Oncol. 2023 May 16;13:1152123. doi: 10.3389/fonc.2023.1152123 (PMC10227592; doi:10.3389/fonc.2023.1152123)
Supplement: Supplementary file 1 [file DataSheet_1.pdf]

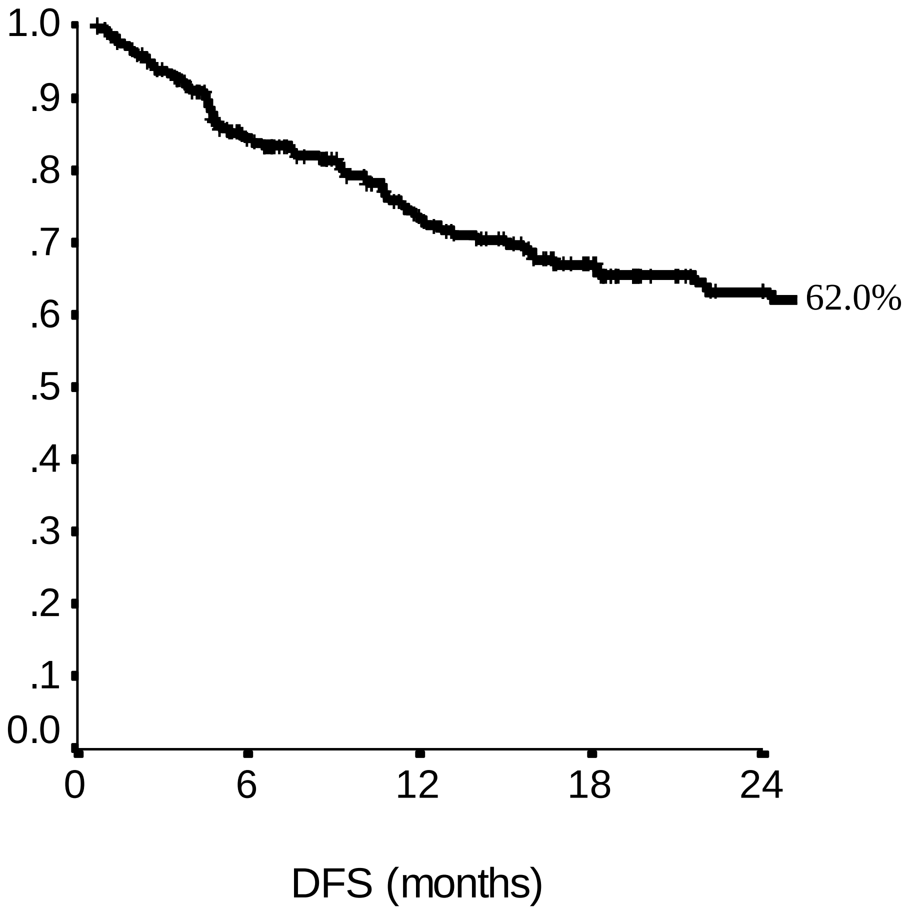

270 178 120 79 24

Supplementary figure 1: Disease free survival in 270 patients

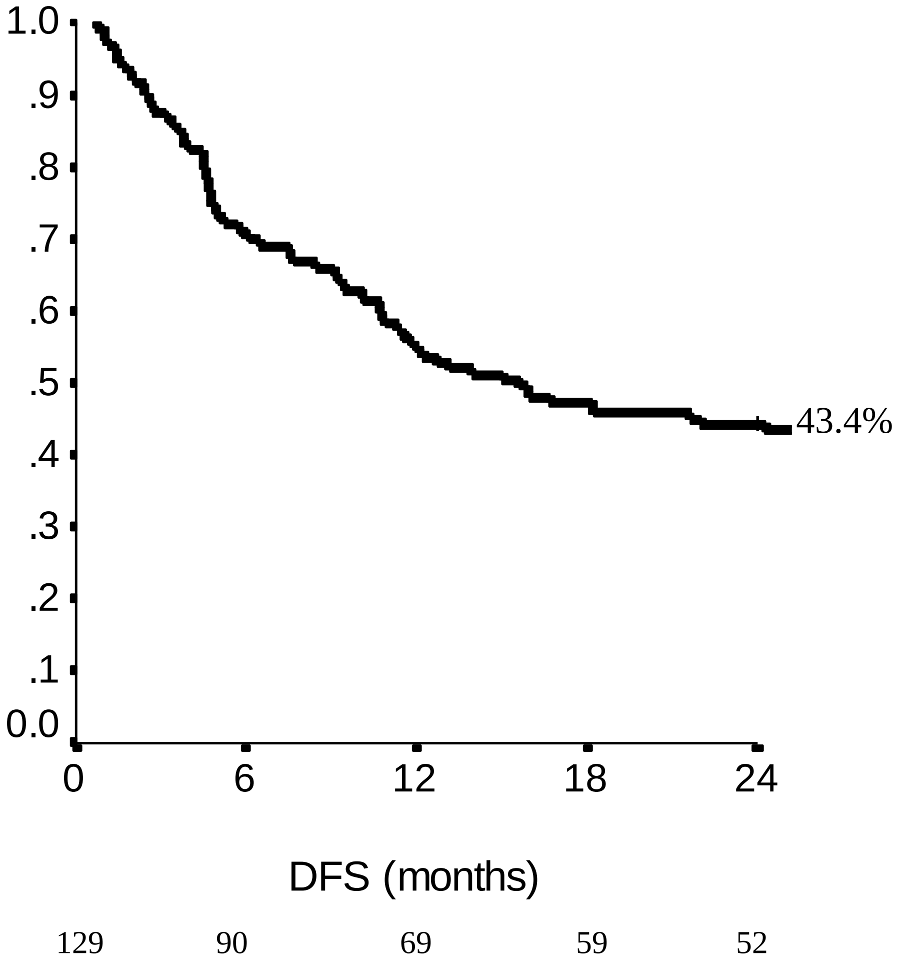

Supplementary figure 2: Disease free survival in 129 patients

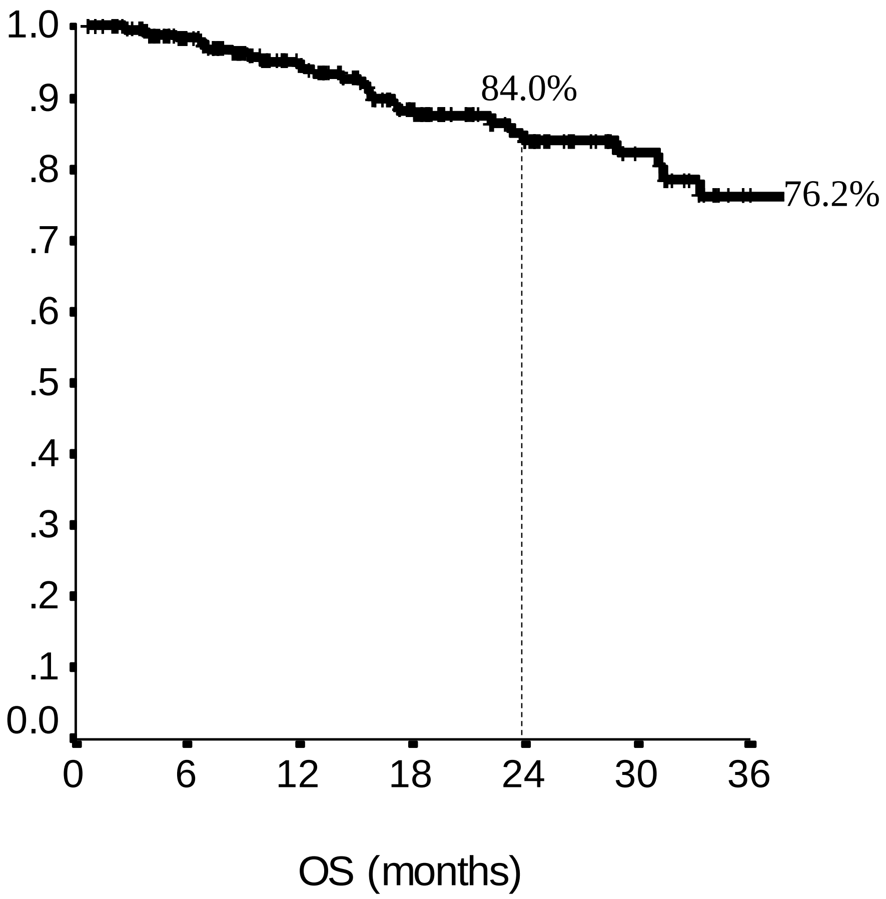

270 203 151 101 66 44 24

Supplementary figure 3: Overall survival in 270 patients

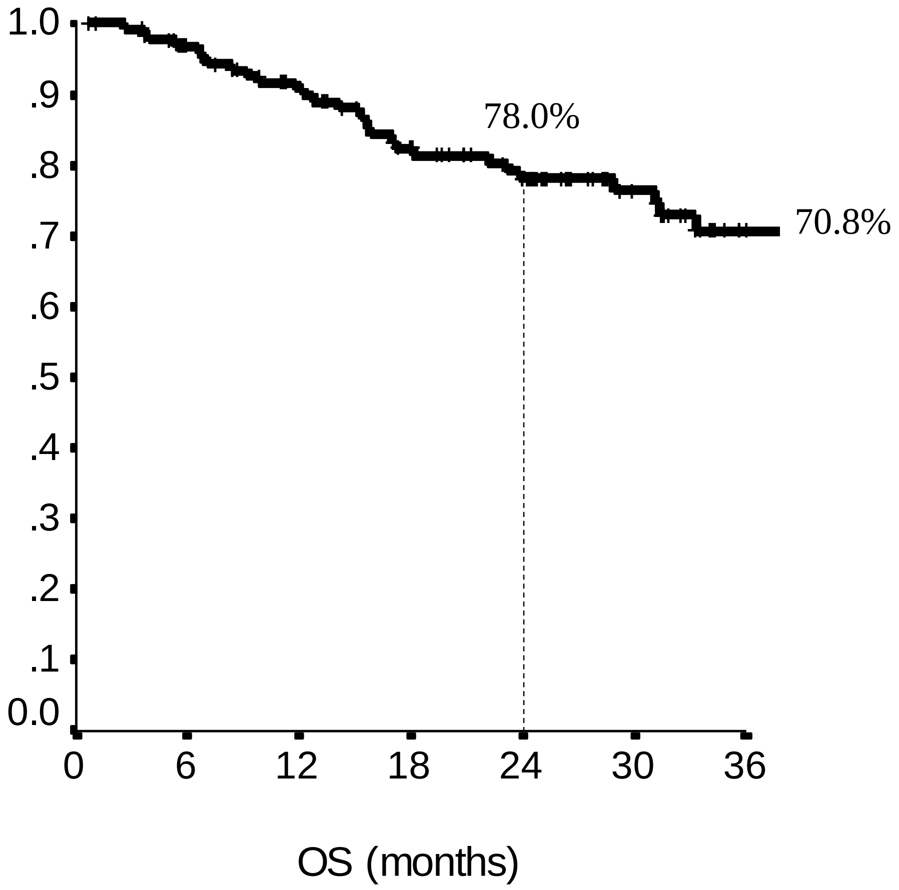

129 115 100 81 66 44 24

Supplementary figure 4: Overall survival in 129 patients

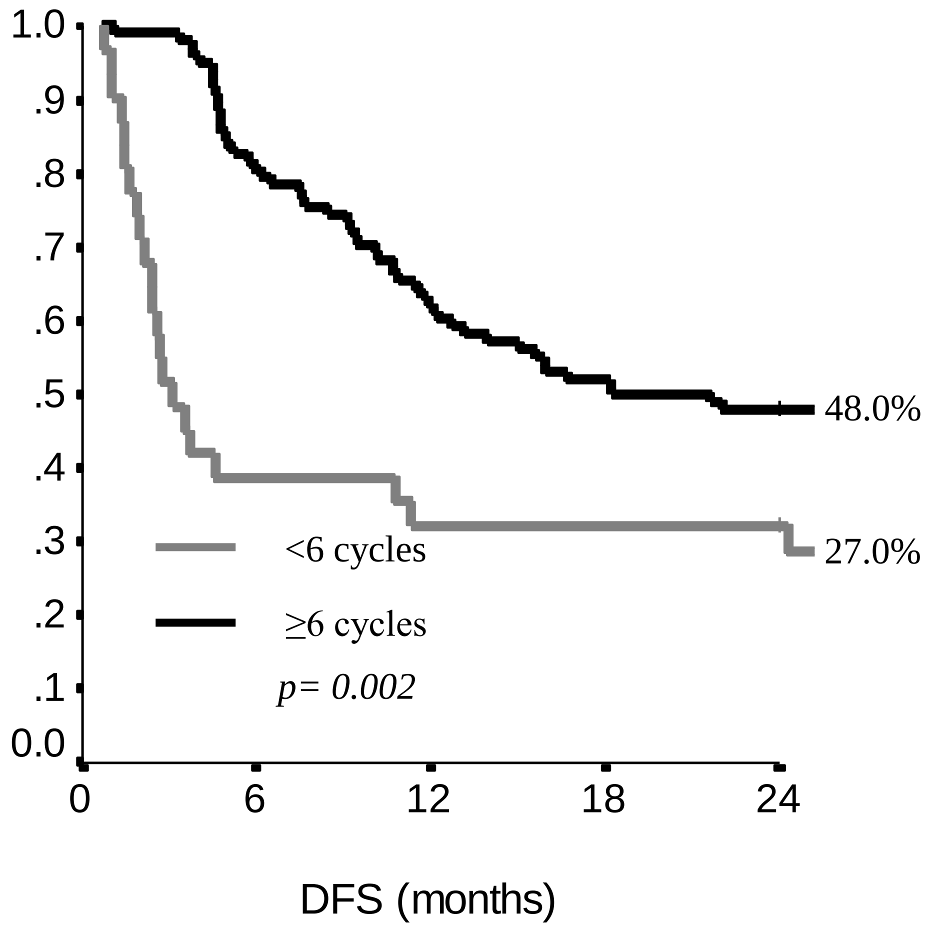

67	21	16	11	8
203	157	104	68	44

Supplementary figure 5: Disease free survival according to capecitabine cycles in 270 patients

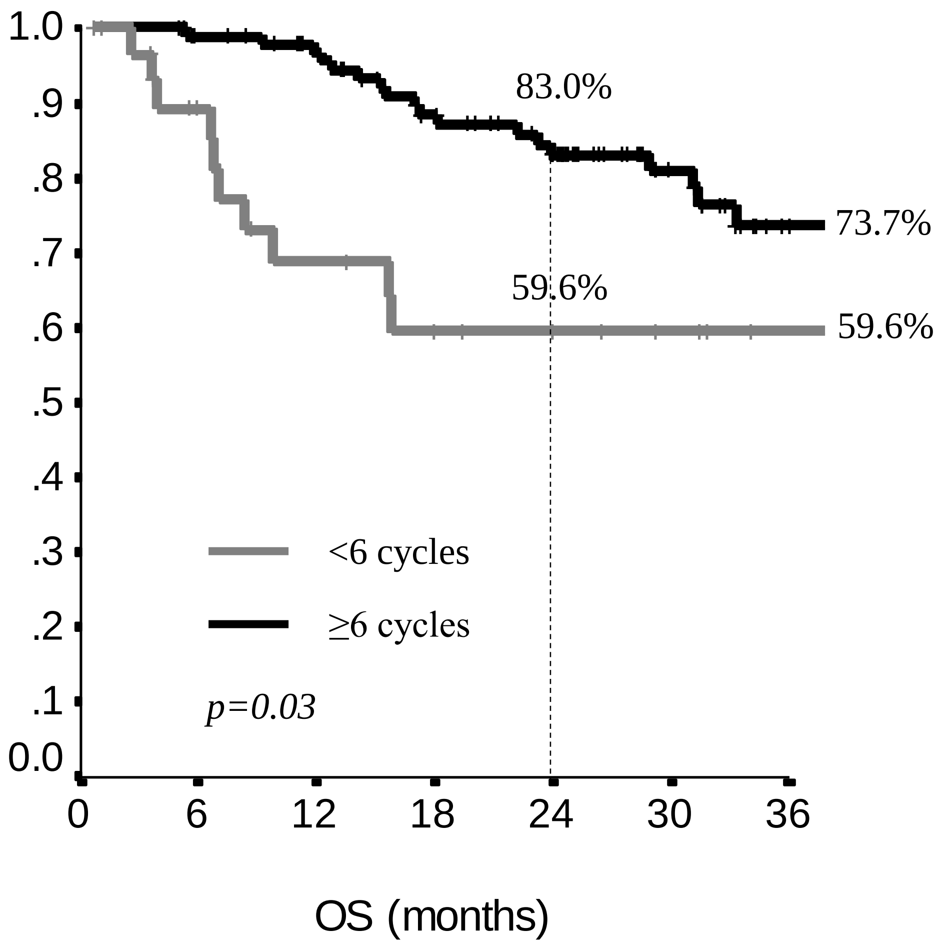

67	31	22	13	10	8	5
203	172	129	88	56	36	19

Supplementary figure 6: Overall survival according to capecitabine cycles in 270 patients
